# The Importance of SiC in the Process of Melting Ductile Iron with a Variable Content of Charge Materials

**DOI:** 10.3390/ma13051231

**Published:** 2020-03-09

**Authors:** Krzysztof Janerka, Łukasz Kostrzewski, Marcin Stawarz, Jan Jezierski

**Affiliations:** 1 Department of Foundry Engineering, Silesian University of Technology, 7 Towarowa, 44-100 Gliwice, Poland; krzysztof.janerka@polsl.pl (K.J.); marcin.stawarz@polsl.pl (M.S.); 2 LFP Ltd., 15 Fabryczna, 64-100 Leszno, Poland; lukasz.kostrzewski.czwa@gmail.com

**Keywords:** ductile iron, silicon carbide, cast iron structure, crystallization

## Abstract

The article presents issues related to melting ductile iron grade EN-GJS-400-15, with different proportions of feedstock (steel scrap and pig iron). The main attention was paid to determining the impact of silicon carbide on the structure and properties of melted cast iron. In the conducted melts, carbon and silicon deficiencies were supplemented with a suitably chosen carburizer, ferrosilicon, and SiC metallurgical silicon carbide. The percentage of silicon carbide in the charge ranged from 0 to 0.91%. The basic condition for the planning of melts was to maintain the repeatability of the chemical composition of the output cast iron and cast iron after the secondary treatment of liquid metal with various charge compositions. Based on the tests, calculations, and analyses of the results obtained, it was concluded that the addition of SiC may increase the number and size of graphite precipitates. Increasing the SiC content in the charge also caused a change in the solidification nature of the alloy and the mechanism of growth of spheroidal graphite precipitates, causing their surface to form a scaly shell. The influence of the addition of silicon carbide on the reduction of the temperature of liquidus in the alloys was also observed. Silicon carbide had a positive effect on the structure and properties of melted alloys. The introduction of SiC into the melting in the studied range caused an increase in the content of carbon and silicon without causing an increase in the amount of impurities in the alloy.

## 1. Introduction

Striving to improve the quality of castings results in a constant search for new solutions. One way is to change the proportion of charge materials used (reducing or eliminating pig iron in the charge and replacing it with much cheaper steel scrap). However, it is connected with the necessity to supplement the deficiency of carbon and silicon in the chemical composition of the melted cast iron. Janerka et al. conducted research on the process of ductile iron melting exclusively based on steel scrap in laboratory conditions [1,2]. They proved that it is possible to produce high-quality ductile iron without pig iron, with proper selection of the carburizing material and the method of carburizing. In this research, they also proved that the carburizing material has a significant impact not only on efficiency and rate of carburizing, and consequently on carburizer yield, but also on the properties and structure of the melted cast iron [3,4]. Supplementation of liquid cast iron with carbon and silicon, resulting from limiting the content of pig iron in the charge, can be conducted sequentially by carburizing and introducing appropriate additives (e.g., FeSi). This process can also be carried out simultaneously by introducing metallurgical silicon carbide. Silicon carbide is currently most frequently used in melting of cast iron in cupola furnaces in the form of briquettes. In induction furnaces, its use is much smaller. Edalati K. et al. melted gray cast iron in an induction electric furnace with a capacity of 25 kg, adding SiC or FeSi to melting. They found that the addition of SiC to the cast iron caused an increase in the amount of class A graphite and its more homogeneous distribution in comparison with FeSi [5]. Stojczew A. et al. melted gray cast iron in an induction furnace with a capacity of 20 kg exclusively based on steel scrap. Deficiency of carbon and silicon was supplemented with FeSi, SiC, and synthetic graphite. Based on the research, they confirmed that cast iron melted with silicon carbide contains thicker graphite precipitations and distributes more evenly than with FeSi [6]. Vasko A. melted ductile iron in an induction electric furnace with a capacity of 30 kg, changing the proportion of pig iron and steel scrap in the charge. Carbon deficiency was supplemented by SiC or FeSi and a carburizer. On the basis of the conducted research, he stated that with an increased share of steel scrap, addition of SiC causes an increase in the number of eutectic grains and, at the same time, reduces probability of occurrence of carbides in the casting structure [7]. Edalati K. and Vasko A., as well as Yunes Rubio R., in their works, state that the addition of SiC increases the temperature of solidus and liquidus. In addition, it increases the number of eutectic grains and, at the same time, reduces the likelihood of carbides in the casting structure. It also reduces the likeliness of microporosity in the casting [7,8,9]. The literature analysis also shows that cast iron, melted on the basis of steel scrap and with appropriately selected carburizing materials, as well as introducing silicon into the metal bath, has much fewer impurities than the cast iron melted using pig iron. This cast iron has much less sulfur and phosphorus and, consequently, fewer slag impurities (fayalite, forsterite). The research on gray and ductile cast iron presented above was performed in laboratory furnaces with a capacity of 20–30 kg. This article describes the results of investigations carried out in the industrial furnace with a capacity of 2300 kg during melting of ductile iron, ensuring better stabilization of the whole process. The novelty of the work is that the experiments were carried out in industrial conditions of melting the cast iron in electric induction furnace. All previous researches with SiC addition into grey and ductile iron were conducted in the laboratory conditions and small melt mass.

## 2. Materials and Methods 

The research included the melting of ductile iron at different contents of charge materials (pig iron, steel scrap, and own scrap). Silicon deficiency was supplemented with FeSi75 ferrosilicon or SiC metallurgical silicon carbide, and the carbon deficiency was corrected with synthetic graphite. The tests were conducted in a network frequency induction furnace with a crucible capacity enabling melting of up to 2300 kg of liquid cast iron. The list of charge materials for completed melts is given in Table 1.

The following were used for melting:

- special pig iron (Pig) containing: 3.5%–4.5% C, 0.5%–1.0% Si, 0.05% Mn, 0.05% P, and 0.02% S,
- steel scrap 1 (Scrap1) of railway tracks with a content of 0.62%–0.80% C, 0.15%–0.58% Si, 0.70%–1.20% Mn, 0.008%–0.025% S, and 0.025% P,
- steel scrap 2 (Scrap2) in the form of compacted deep-drawing sheet waste with 0.028% C, 0.009% Si, 0.025% Mn, 0.020% S, and 0.020% P,
- own cast iron scrap/returns (Ret) from ductile iron grade EN GJS 400-15,
- metallurgical silicon carbide (SiC) containing: 85% SiC, 1.5%–2.5% C, 4.58% Si, 1%–2% Fe, and 3%–6% SiO_2_,
- FeSi75 ferrosilicon (FeSi) with the chemical composition of: 75.09% Si, 1.49% Al, 0.145% C, 0.026% P, 0.007% S, and 0.77% Ca.

Carburizer (Carb) in the form of synthetic graphite with granulation of 0.5–4 mm and content: Cmin—94%, Smax—max. 0.1%, ash content—max. 2%, humidity—max. 1%, and volatile matter content—max. 1%.

Due to the fact that the research process was carried out in the production cycle, it was assumed that the mass of silicon carbide introduced would not exceed 1%. It meant adopting recommendations contained in The Sorelmetal book of ductile iron, stating that excess silicon carbide can cause accelerated wear and tear of the furnace lining and formation of adhesions on the furnace walls [10]. After melting the charge and supplementing the required alloying elements, the sample for chemical composition tests was cast and the probe for thermal analysis was poured over. Next, the metal from the furnace was poured in portions into the treatment ladle, where spheroidization and inoculation were carried out. Spheroidization was carried out using the Sandwich method with FeSiMg6Ce master alloy. The inoculant (SB 5) was introduced during the transfer of liquid metal from the process ladle after the spheroidization procedure to the pouring ladle. After these operations, a sample was taken for chemical analysis and ingots were cast for strength tests, in accordance with EN 1563:2011.

The tests included measurements of tensile strength and hardness. For this purpose, separate Y-test ingots were cast. For tensile strength tests, samples with a diameter of 14 mm and a length of 84 mm, with threaded grips, were used. Hardness measurements were made using the Brinell method. Metallographic tests were performed on samples cut from test ingots.

## 3. Results and Discussion

The results of the chemical analysis of the base cast iron before the spheroidization process are presented in Table 2. This table also contains the results of thermal analysis, where the following determinations were made: TL—liquidus temperature, TS—solidus temperature, CE—eutectic carbon equivalent, Sc—eutectic saturation factor. During each melt, the cooling curves were captured with use of QuiK-Lab E measuring system. The thermal analysis is a quick and cheap method of direct liquid iron quality control [11]. The characteristic values of TL and TS were registered for each TA test. The cups of QuiK-Cup type QC4010 (without Te addition) were used.

The carbon content in the base cast iron varied in the range of 3.72%–3.94% and silicon content in the range of 1.34%–1.73%. Low sulfur content (0.007%–0.020%) caused lower consumption of magnesium master alloy, while low manganese content (0.16%–0.30%) guaranteed obtaining cast iron with a predominance of ferritic matrix. 

**Figure 1:**
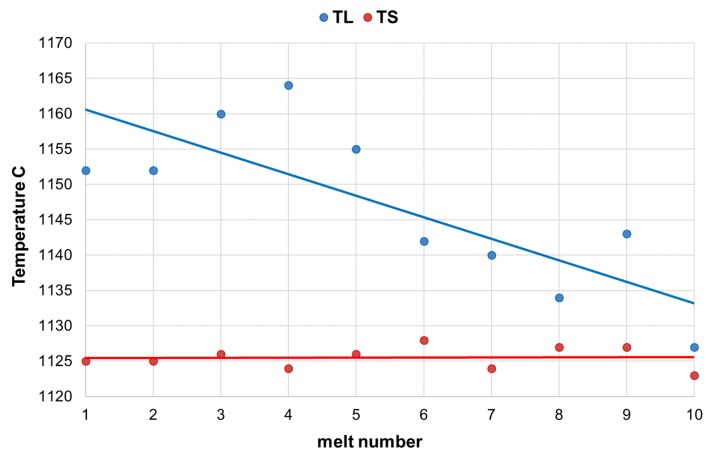
Impact of SiC content (increase of additive in subsequent melts) on liquidus TL and solidus TS temperature.

Analyzing the solidification process of the examined alloys, a decrease in liquidus temperature can be seen, along with an increase in the SiC content in the furnace charge. At the same time, very repeatable solidus temperature readings were found in these melts. This resulted in the narrowing of the crystallization temperature range (Figure 1). It positively affected the number and quality of graphite precipitates, which was also observed during the quantitative and qualitative analysis of the microstructure of the researched alloys, presented in the further part of the publication.

In turn, the results of chemical analysis after the spheroidization process are presented in Table 3.

Analyzing the chemical composition of melted cast iron, it was found that a very small range of variability of individual elements was obtained despite the different proportions of the charge components. Very low content of sulfur and phosphorus was obtained in all melts, which is a consequence of the low content of these elements in both pig iron and steel scrap. Analyzing the content of trace elements (B, Cr, V, Ti, As) not included in the tables, and which may appear in the melting, e.g., as a result of the introduction of SiC, no increase was noted in relation to the melting when using pig iron.

In the case of ferritic cast iron, steel scrap is particularly important, as it must have a very low manganese content. Studies have shown that appropriate scrap and its proper selection allow for obtaining the Mn content in the melt even below 0.2%, which in combination with low Cu, Sb, and Ni content, as well as adequate Mg content, ensures ferritic structure. The content of residual magnesium in the melt changing in the range of 0.042%–0.058% ensured obtaining very high-quality ductile iron.

The obtained measurement results for mechanical properties are presented in Table 4, where the following determinations were adopted: UTS—ultimate tensile strength, YS—yield strength, e—elongation, BHN—Brinell hardness number.

**Figure 2:**
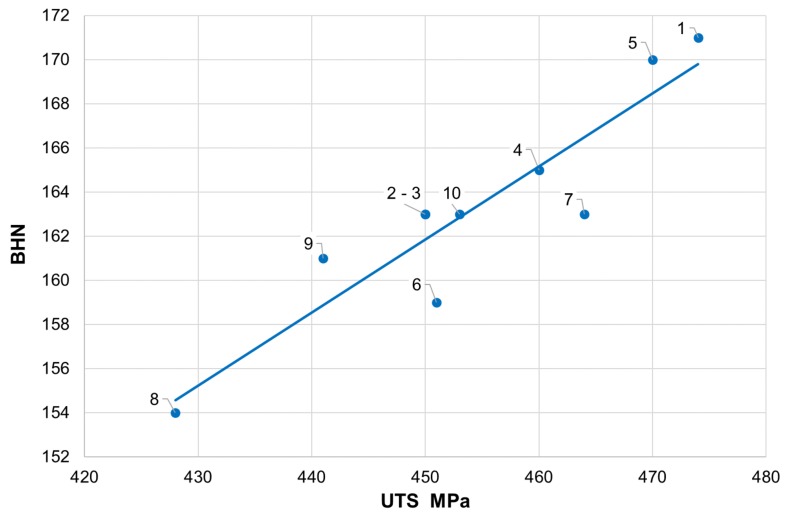
Ultimate tensile strength (UTS) and Brinell hardness number (BHN) in individual melts.

When analyzing the mechanical properties of cast iron, it should be stated that all measured parameters exceed the values given in the standard, where for cast iron grade GJS-400-15, the minimum UTS = 400 MPa and minimum elongation = 15%. This applies to tensile strength (428–474 MPa), the yield strength (287–329 MPa), and elongation (18%–24%). The hardness obtained is also high in terms of strength (Figure 2).

**Figure 3:**
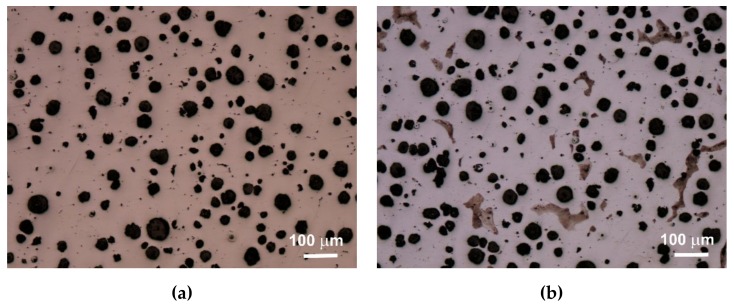
Microstructure of sample 1 non-etched (**a**) and etched (**b**).

**Figure 4:**
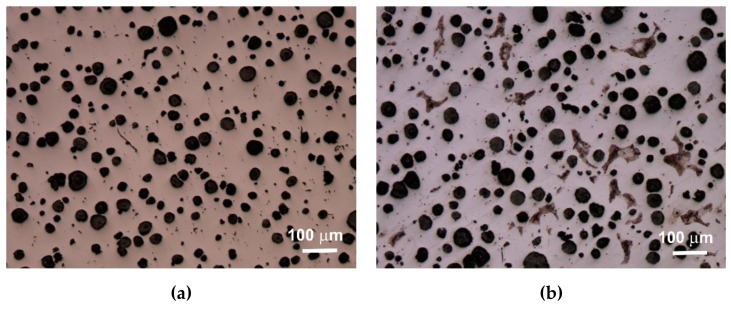
Microstructure of sample 5 non-etched (**a**) and etched (**b**).

**Figure 5:**
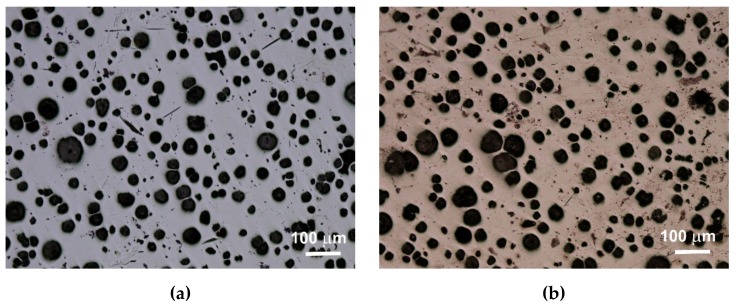
Microstructure of sample 9 non-etched (**a**) and etched (**b**).

An extremely important issue in the assessment of ductile iron is the analysis of its microstructure. This applies to both the graphite precipitates and the matrix [12,13]. The consequence of the number of graphite precipitates and their shape, as well as the different amounts of ferrite and pearlite in the structure, are specific strength properties. Apart from the chemical composition of melted cast iron, the structure is determined by heat dissipation conditions, depending on the shape of the casting and the molding sand used. Examples of etched and non-etched microstructure images at 100x magnification for some of the melts are shown in Figure 3, Figure 4 and Figure 5. Samples were etched with 2% nital.

Quantitative analysis was carried out for the sections made. Image analysis was carried out using the Nikon NIS-BR program. The analysis was carried out according to ISO 945-1:2017 and the microstructure parameters were analyzed according to ASTM E2567-16a as it was presented in Reference [12]. Its results are presented in Table 5.

**Figure 6:**
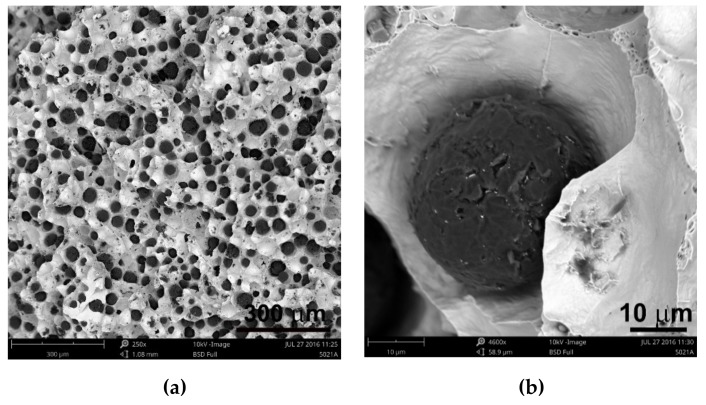
Images of sample fractures 1 magnification 250× (**a**) and 4600× (**b**).

**Figure 7:**
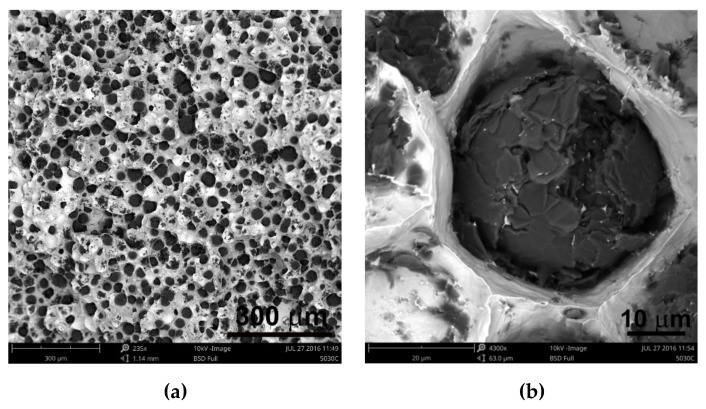
Images of sample fractures 5 magnification 235× (a) and 4300× (**b**).

**Figure 8:**
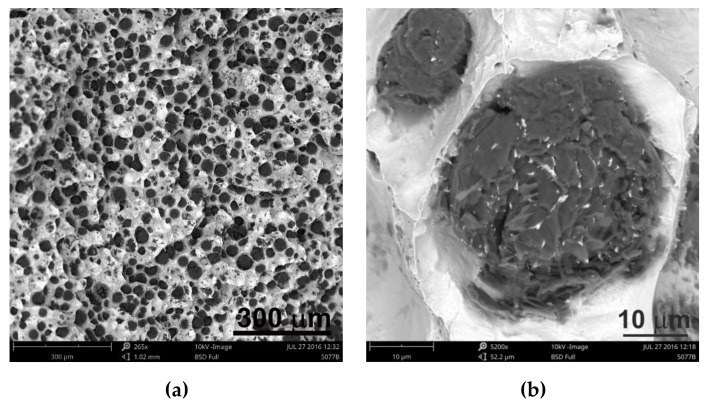
Images of sample fractures 10 magnification 265x (a) and 5200x (b).

Analyzing the obtained results of measurements and calculations, it can be seen that the number of graphite precipitates per mm^2^ was between 300 and 407. It is estimated that for the ductile cast iron minimum 80% of the graphite precipitations should fit the range of the shape factor (roundness) 0.8–1.0. The shape factor was above 0.938 and it was very high. Only in the case of cast iron based on pig iron, the value of this parameter is 0.886. For some of the melts, observations of sample fractures were performed using a scanning electron microscope (Figure 6, Figure 7 and Figure 8).

Analysis of fractures of chosen samples under a scanning electron microscope shows similar morphological features of the observed graphite precipitates. Subsequent layers of accruing graphite flakes are relatively large, which makes the surface of the nodules smooth. For sample 1 (without the addition of SiC), the accruing layers of graphite flakes are smaller in size, forming a scaly shell on the surface of the spheroid precipitate. When FeSi and SiC are introduced into cast iron, a less regular surface with protruding flakes appears on the surface of the graphite balls and a much larger number of cracks in these nodules is observed.

Undoubtedly, in these cases, there are various mechanisms of graphite growth, which were described by Stefanescu D.M. et al. [14], who stated that the basic elements forming graphite aggregates are hexagonal graphite plates generated by growth of graphene layers. As the solidification progresses, the plates thicken to form successive layers. They developed this theory in their work [15], proving that these plates are built according to various mechanisms: tile, curved-circumferential, spiral, and pyramidal or conical. Stefanescu D.M. et al. stated that the final shape of graphite spheroids is influenced by nuclear crystallography because it determines the initial growth of graphite plates [15].

Searching for the reasons for these differences, the authors of this publication propose two hypotheses to explain these differences.

The first hypothesis is that the reason for differences in structure may be SiC, the greater content of which causes a change in growth parameters, and in particular its inhibition in the main crystallographic directions. Due to the influence on crystallization conditions, it is possible to lead to different growth variants of polycrystalline graphite precipitates, underlying which there are phenomena of incorporation of elements inhibiting graphite growth in crystallographic directions [1010], formation of dislocations, etc. The addition of SiC alloy certainly affects these conditions.

The second hypothesis is based on previous research conducted by Janerka K. et al. consisting of carburizing liquid cast iron with various graphite and non-graphite carburizing materials in an electric furnace with a capacity of 20 kg. They found that the main structural elements of the carburizers are fragments of two-dimensional hexagonal networks (graphite crystallites) arranged in parallel at a distance of approximately 0.335 nm [4]. This confirms the theory developed and described by Fitzer E. et al. [16] for well-arranged carbon materials. Inappropriately arranged carbon materials are composed of structural units built of packets of several graphite layers. They have a diameter of several nanometers and are arranged approximately in parallel. Distances between them are much greater than in case of graphite. They lack arrangement in the direction perpendicular to their surface, as well as correlation of the atom position in adjacent layers. The elements are an intermediate form between a crystalline (graphite) and an amorphous form and they are called “turbostatic crystallite”. These issues have been presented in more detail by Oberlin A. in Reference [17]. Carburization is also present when introducing SiC into the cast iron. Perhaps, in this case, graphene layers are not arranged in parallel but randomly. This causes subsequent layers to grow, not according to the tile model, but spirally, pyramidally, or conically, which in turn results in the jagged surface of most spheroidal precipitates.

## 4. Summary and Conclusions

As part of implementing the research, melting of ductile iron was carried out for various proportions of charge materials (pig iron and steel scrap) and additives (carburizer, FeSi, SiC). The mass of silicon carbide introduced varied in the range of 0–20 kg, which constituted 0–0.91%. The authors stated that only up to 1% changes in the microstructure of the researched alloys can be observed and, above this content, the effect associated with the addition of SiC is not proportional to the increase in the amount of added material [10]. 

Analyzing the charge structure, it can be seen that ferritic cast iron was melted without the special pig iron in the charge and with its 10%, 20%, and 55% content. The chemical analysis, tests of strength properties and microstructure showed that in all melts the ductile iron was obtained that met the assumed requirements. 

Based on images of microstructures obtained under an optical microscope, it was found that in all cases the spheroid precipitates of graphite were obtained. These precipitates are evenly distributed. The cast iron matrix is ferritic. Based on the quantitative analysis of the structures, it was found that the number of precipitates per 1 mm^2^ was 300–407, and the shape factor was 0.886–0.954. The ferrite content varied from 92.07% to 99.70%. The analysis of the shape factor distribution allows to state that in the case of melts 1 and 2 (based on pig iron, without SiC), much fewer precipitates in the coefficient class equal to 1.0 (30% and 45%) were obtained, while in other melts this value was 62%–69%. Based on the presented results of the quantitative analysis of the structure, it can be hypothesized that increasing the amount of SiC in the charge causes an increase in the number of graphite precipitates and they are larger, which results in a greater content of graphite.

Based on the tests, calculations, and analyses of the results obtained, the following conclusions can be drawn:

1. It is possible to melt ductile ferritic and pearlitic-ferritic cast iron without pig iron in the charge while maintaining its high mechanical properties and proper structure.
2. The introduction of SiC into the melt in the studied range (up to 0.91%) causes an increase in the content of carbon and silicon without causing an increase in the amount of impurities in the alloy.
3. Increasing the SiC addition in the charge may result in an increase in the number and size of graphite precipitates.
4. The addition of SiC to the alloy changes the solidification nature of the alloy and affects the mechanism of growth of spheroidal graphite precipitates. In the conducted studies, this effect was observed in the form of changes in the structure of the surface layer of spheroidal graphite precipitates, whose morphology clearly differs from graphite precipitations of alloys without the addition of SiC.
5. A definite influence of the SiC addition on the reduction of liquidus temperature in the tested alloys was also noted.

## Figures and Tables

**Table 1:** List of charge materials and additives.

Melt No.	Charge Material				Additive		
	Pig, kg	Scrap 1, kg	Scrap 2, kg	Ret, kg	Carb, kg	FeSi, kg	SiC, kg
1	1200	200	-	700	-	-	-
2	1200	200	-	700	-	-	-
3	200	-	900	1050	50	8	-
4	200	-	900	1050	50	5	-
5	200	-	900	1100	40	8	10
6	200	-	900	1100	40	-	10
7	200	-	900	1100	35	-	15
8	200	-	900	1100	35	-	15
9	-	-	1200	1000	45	-	20
10	-	-	1200	1000	45	-	20

**Table 2:** Results of chemical and thermal analysis of the base cast iron.

Melt No.	C, %	Si, %	Mn,%	P, %	S, %	Cr, %	Cu, %	TL	TS	CE	Sc
1	3.85	1.54	0.26	0.027	0.020	0.027	0.05	1152	1125	4.19	1.024
2	3.88	1.35	0.19	0.040	0.007	0.023	0.02	1152	1125	4.19	1.024
3	3.72	1.43	0.24	0.027	0.020	0.027	0.05	1160	1126	4.12	1.005
4	3.83	1.49	0.24	0.036	0.014	0.027	0.04	1246	1124	4.38	1.072
5	3.84	1.54	0.28	0.026	0.018	0.034	0.12	1155	1126	4.17	1.017
6	3.87	1.47	0.25	0.031	0.012	0.028	0.09	1142	1128	4.28	1.047
7	3.94	1.59	0.30	0.038	0.019	0.032	0.10	1140	1124	4.44	0.990
8	3.83	1.41	0.16	0.022	0.014	0.024	0.03	1134	1127	4.18	1.022
9	3.90	1.34	0.18	0.020	0.018	0.027	0.03	1143	1127	4.27	1.045
10	3.83	1.73	0.19	0.019	0.014	0.037	0.06	1127	1123	4.41	1.081

**Table 3:** Chemical analysis of cast iron after the spheroidization process.

Melt No.	C, %	Si, %	Mn, %	P, %	S, %	Cr, %	Cu, %	Mg, %
1	3.48	2.53	0.25	0.028	0.011	0.031	0.06	0.052
2	3.66	2.64	0.20	0.040	0.009	0.028	0.02	0.052
3	3.52	2.52	0.24	0.028	0.011	0.031	0.06	0.052
4	3.56	2.45	0.24	0.034	0.009	0.032	0.04	0.045
5	3.68	2.66	0.28	0.027	0.012	0.035	0.12	0.058
6	3.71	2.55	0.26	0.031	0.013	0.03	0.09	0.052
7	3.67	2.59	0.29	0.036	0.011	0.036	0.10	0.050
8	3.58	2.44	0.17	0.022	0.012	0.025	0.03	0.047
9	3.72	2.55	0.18	0.020	0.010	0.028	0.03	0.058
10	3.57	2.53	0.19	0.019	0.012	0.038	0.06	0.050

**Table 4:** Test results of mechanical properties.

Melt No.	UTS, MPa	e, %	YS, MPa	BHN
1	474	23.5	336	171
2	450	24	317	163
3	450	24	317	163
4	460	21.5	317	165
5	470	23	329	170
6	451	22	311	159
7	464	24	324	163
8	428	23	287	154
9	441	18	306	161
10	453	20.5	312	163

**Table 5:** Results of the quantitative analysis of the structure (average values).

Melt No.	Number of graphite precipitates per mm^2^	Surface of graphite precipitates, μm^2^	Coefficient of shape	Content of pearlite, %	Content of ferrite, %
1	310	216.4	0.886	4.12	95.88
3	407	182.6	0.938	2.38	97.62
5	300	240.9	0.946	4.51	95.49
6	385	235.8	0.954	3.78	96.22
8	367	240.9	0.945	1.63	98.37
9	384	264.5	0.949	0.30	99.70
